# A low serum iron level is a potential predictor of poor renal function in patients following laparoscopic sleeve gastrectomy: a retrospective study

**DOI:** 10.1038/s41598-021-01608-5

**Published:** 2021-11-15

**Authors:** Tien-Chou Soong, I.-Jung Feng, Jen-Yin Chen, I.-Wen Chen, Hong-Yi Tong, Ming-Yuen Yang, Shu-Fen Wu, Ming Yew, Kuo-Chuan Hung

**Affiliations:** 1 Department of Weight Loss and Health Management Center, E-DA Dachang Hospital, Kaohsiung City, Taiwan; 2 Department of Asia Obesity Medical Research Center, E-DA Hospital, Kaohsiung City, Taiwan; 3 College of Medicine, I-Shou University, Kaohsiung City, Taiwan; 4 Institute of Precision Medicine, National Sun Yat-Sen University, Kaohsiung City, Taiwan; 5 Department of Anesthesiology, Chi Mei Medical Center, Tainan City, Taiwan; 6 Department of General Surgery, Yuan’s General Hospital, Kaohsiung City, Taiwan; 7 Department of Nursing, Shu-Zen Junior College of Medicine and Management, Kaohsiung City, Taiwan; 8 Department of Anesthesiology, Chi Mei Hospital, Liouying, Tainan city, Taiwan; 9 Department of Hospital and Health Care Administration, College of Recreation and Health Management, Chia Nan University of Pharmacy and Science, Tainan City, Taiwan

**Keywords:** Health care, Medical research

## Abstract

This study aimed to assess the association of serum iron level (Iron) with the estimated glomerular filtration rate (eGFR) after bariatric surgery (BS). We reviewed 210 patients with mean age of 39.1 ± 10.6 years (body mass index, 41.4 ± 5.5 kg/m^2^) undergoing BS. The primary outcome was the relationship between Iron and eGFR at 12-month after surgery. Multiple linear regression analyses were performed using postoperative eGFR as dependent variables and using Iron and other variables (i.e., age) as independent variables. At 12-month follow-up, 94 patients were analyzed. BMI significantly decreased, whereas serum iron level significantly increased. Although the percentage of patients with eGFR of < 90 mL/min/1.73 m^2^ increased during the study period, no significant difference was found in postoperative 12-month eGFR. No correlations were noted between Iron and eGFR at baseline and postoperative 1 and 6 months, whereas a significant relationship was observed between Iron and postoperative 12-month eGFR. Multiple linear regression analyses revealed that Iron and presence of diabetes were the independent predictors of postoperative 12-month eGFR. This pilot study showed a positive association of postoperative serum iron level with renal function in this patient population. Further large-scale trials are needed to confirm the findings.

## Introduction

Chronic kidney disease (CKD) with a global prevalence of 13.4% has been reported to increase the risk of cardiovascular disease by 2–4 times, and it is considered a health burden worldwide^1,2^. Current evidence has demonstrated that proteinuria or low estimated glomerular filtration rate (eGFR) is related to progression of renal disease^3^, underscoring the clinical significance of intervention strategies to improve renal function. Obesity is a well-known independent risk factor for CKD development^4,5^. Several meta-analyses have shown that bariatric surgery (BS), which is an efficient intervention for obtaining substantial weight loss, may improve renal function or prevent further decline in renal function in patients with morbid obesity (MO)^6–8^. Nevertheless, postoperative 1-year eGFR either reportedly did not change significantly^9^ or decreased from normal to the range indicating stage 2 CKD (from 91.6 to 82.6 mL/min) after BS^10^.

Currently, there are inconsistent findings regarding the effect of BS on postoperative renal function; therefore, an investigation of potential prognostic factors that may have a positive impact on postoperative renal function is necessary. Although BS improves obesity-related comorbidities (e.g., diabetes), postoperative micronutrient deficiency is common. Iron deficiency is among the most common nutritional deficiencies after BS, with a prevalence of 15%–60% and 30%–43% in patients undergoing gastric bypass^11–14^ and sleeve gastrectomy, respectively^1,15^. Evidence suggests that iron homeostasis is associated with preservation of residual renal function. For instance, elevated serum iron levels reportedly play a critical protective role for renal and liver allografts through immunomodulation^16,17^. Further, systemic iron overload could protect against renal ischemia–reperfusion injury in mice^16^. To explore the potential influence of the serum iron status on postoperative renal function, we aimed to assess the relationship between serum iron level and postoperative 12-month eGFR.

## Materials and methods

### Study design and population

The Institutional Review Board of Yuan’s General Hospital reviewed the protocol and procedures of the current study as well as approved the waiving of informed consent in view of the retrospective design (Approval No. 20201111B). This study was performed in accordance with the Declaration of Helsinki. This retrospective study was conducted on patients with MO who underwent laparoscopic sleeve gastrectomy (LSG) from 2014 to 2017 at an academic center. The recruitment criteria included age of ≥ 20 years and BMI of ≥ 35 kg/m^2^. Conversely, those with severe cardiopulmonary disease (e.g., heart failure), a history of alcohol/substance addiction, an American Society of Anesthesiologists score of > 3, glomerular hyperfiltration (eGFR ≥ 125 mL/min/1.73 m^2^), and missing laboratory data; those undergoing dialysis; those undergoing previous gastric surgery or revision surgeries; and those who died or were lost to follow-up during the study period were excluded.

### Preoperative laboratory examination and surgical procedures

Esophagogastroduodenoscopy was preoperatively used to diagnose possible infection with Helicobacter pylori and peptic ulcers, which would be treated preoperatively. Additionally, serum biochemistry profiles including triglyceride, aspartate aminotransferase, alanine aminotransferase, total cholesterol, low- and high-density lipoproteins, complete blood count, and fasting glucose levels were determined. Height and body weight were measured simultaneously. BS including laparoscopic Roux-en-Y gastric bypass, laparoscopic adjustable gastric banding, and LSG is offered at our institute. Among these surgeries, > 90% of procedures included LSG, and all surgeries were performed by the same surgeon. Regarding LSG, the greater curvature was devascularized from the gastroesophageal junction to the pylorus. A 38-Fr orogastric tube was introduced through the esophagus to indicate the extent of gastric resection. By using a single 3–0 ethibond suture, the stomach and retroperitoneal tissue were fixed in place to avoid gastric torsion.

### Postoperative nutritional counseling and supplements

During postoperative 1 year, follow-ups were performed at postoperative 1, 2, 3, 6, 9, and 12 months. At every visit, physical examination and weight loss (WL) were documented. Although examinations of serum biochemistry profiles and nutritional status were recommended, they were not mandatory at every visit. Moreover, a qualified dietitian offered routine nutritional counseling to help patients decrease the risk of subsequent nutritional deficiencies. To ensure compliance with the policy followed at our institute, each patient was daily administered two commercial chewable multivitamin/mineral preparations [each containing calcium (104 mg); folic acid (200 µg); zinc (7.5 mg); and vitamins A, B12, E, D, and B1 (488, 100, 83, and 7.5 µg and 5 mg, respectively)]. Daily oral elemental iron (24 mg) was prescribed when a low serum iron level (serum iron level < 60 µg/dL)^15^ was noticed.

### Definitions and outcomes

Percent WL (%WL) was determined by dividing the amount of WL by the patient’s preoperative weight. Impaired renal function was defined as an eGFR of < 90 mL/min/1.73 m^2^ measured using the Chronic Kidney Disease Epidemiology Collaboration equation^18^. The primary study endpoint was the relationship between serum iron level and postoperative 12-month eGFR. The secondary endpoints were renal function and serum iron level changes at different postoperative timepoints.

### Statistical analysis

To determine a correlation between serum iron level and postoperative renal function (i.e., eGFR) of r = 0.3 (α error of 5% and power of 80%), a sample size of 64 patients was required. Chi-square test was used to compare categorical variables (frequencies and percentages), and continuous variables are presented as means and standard deviations. Student’s t-test and Mann–Whitney U-test were used to compare normally and non-normally distributed continuous variables, respectively. Normality of variables was investigated with the Kolmogorov–Smirnov test. Pearson’s or Spearman’s correlation was applied to investigate the relationship between serum iron level and postoperative eGFR where appropriate.

Multiple regression analyses were carried out using serum creatinine level or postoperative eGFR as dependent variables and using serum iron levels as well as other variables (e.g., age or postoperative 1-year BMI) as independent variables. The variance inflation factor was used to detect possible collinearity or autocorrelation of independent variables in this model. In addition, multicollinearity was considered significant when the variance inflation factor was > 10. A receiver operating characteristic (ROC) curve was plotted to determine the optimal cutoff value of serum iron levels for predicting postoperative eGFR of ≥ 90 mL/min/1.73 m^2^ in subgroup patients with normal renal function at baseline. The optimal cutoff value was determined using Youden’s index by maximizing the point on the ROC curve furthest from the line of equality. Statistical analyses were performed using the Statistical Package for the Social Sciences (version 22.0; Chicago, IL). Statistical significance level was set at 0.05.

### Institutional review board statement

The study was conducted according to the guidelines of the Declaration of Helsinki, and approved by the Institutional Review Board of Yuan’s General Hospital (protocol code 20201111B; date of approval: January 20, 2021).

### Informed consent

Patient consent was waived due to retrospective study design.

## Results

### Patient demographics and anthropometric parameters

**Figure 1:**
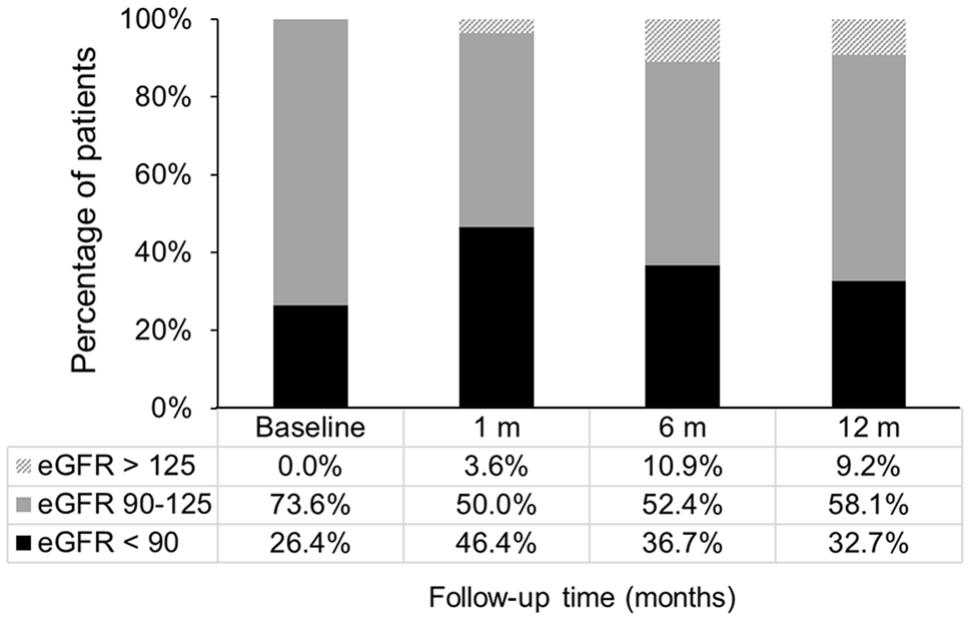
Incidence of renal impairment, defined as estimated glomerular filtration rate (eGFR) of < 90 mL/min/1.73 m^2^, at baseline, 1-, 6-, and 12-month following laparoscopic sleeve gastrectomy.

**Figure 2:**
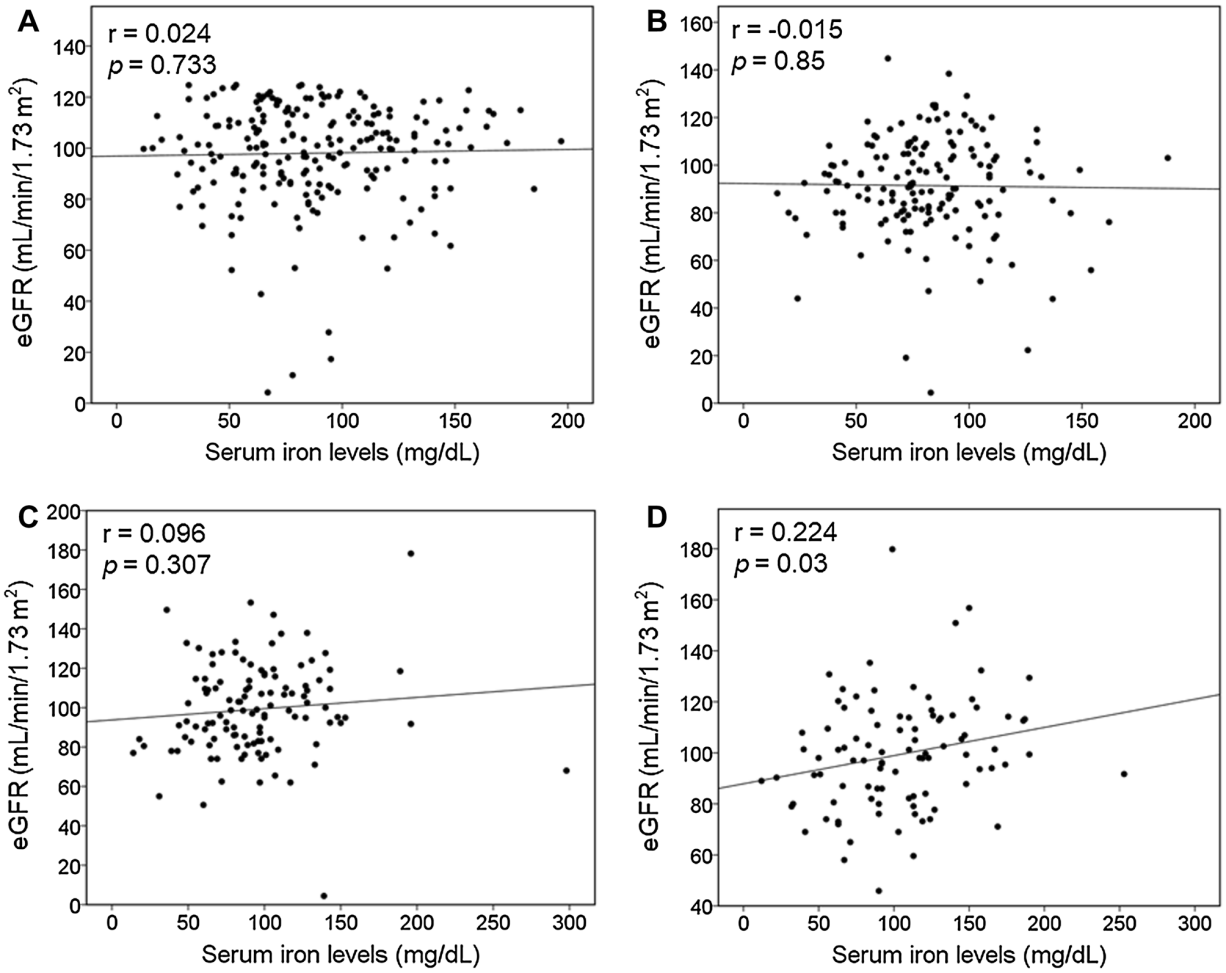
The associations of serum iron concentrations with renal function (i.e., estimated glomerular filtration rate) at (**A**) baseline and (**B**) 1, (**C**) 6, and (**D**) 12 months following laparoscopic sleeve gastrectomy.

During the study period, a total of 316 patients with obesity undergoing LSG were retrospectively reviewed. Seventy-eight patients were excluded because of eGFR ≥ 125 mL/min per 1.73 m^2^ (n = 41), American Society of Anesthesiologists score of > 3 (n = 25), missing data (n = 19), history of dialysis (n = 12), and revision surgeries (n = 9). In total, 210 patients (mean age, 39.1 ± 10.6 years) were included (Table 1). Among these, 26.4% had impaired renal function at baseline (Fig. 1). All patients received surgical procedures and medical care by the same surgeon and medical team. Table 1 summarizes the demographic characteristics, serum iron concentrations, and comorbidities of patients receiving LSG. The mean preoperative BMI was 41.4 ± 5.5 kg/m^2^ (range 35.0–72.3 kg/m^2^). Further, the mean preoperative serum iron levels were 87.4 ± 36.3 µg/dL with an incidence of low serum iron level of 21.4%. At baseline, no significant association of BMI with eGFR (r =  − 0.047; *p* = 0.491) and serum iron levels (r =  − 0.086; *p* = 0.215) was noted. eGFR was also not related to serum iron levels at baseline (r = 0.024; *p* = 0.733) (Fig. 2A).

**Table 1:** Demographic characteristics of patients undergoing laparoscopic sleeve gastrectomy.

Variables	Study population (n = 210)
Age (year)	39.1 ± 10.6
Female (%)	122 (55.5%)
Height (cm)	166.0 ± 8.6
Weight (kg)	114.4 ± 20.1
Body mass index (kg/m^2^)	41.4 ± 5.5
Diabetes mellitus, n (%)	48 (21.8%)
Serum iron concentration (µg/dL)	87.4 ± 36.3
Patients with low serum iron (< 60 µg/dL)	45 (21.4%)

Data are presented as mean ± standard deviation or as total number of patients (%).

### BMI, metabolic profiles, and renal function after sleeve gastrectomy

**Figure 3:**
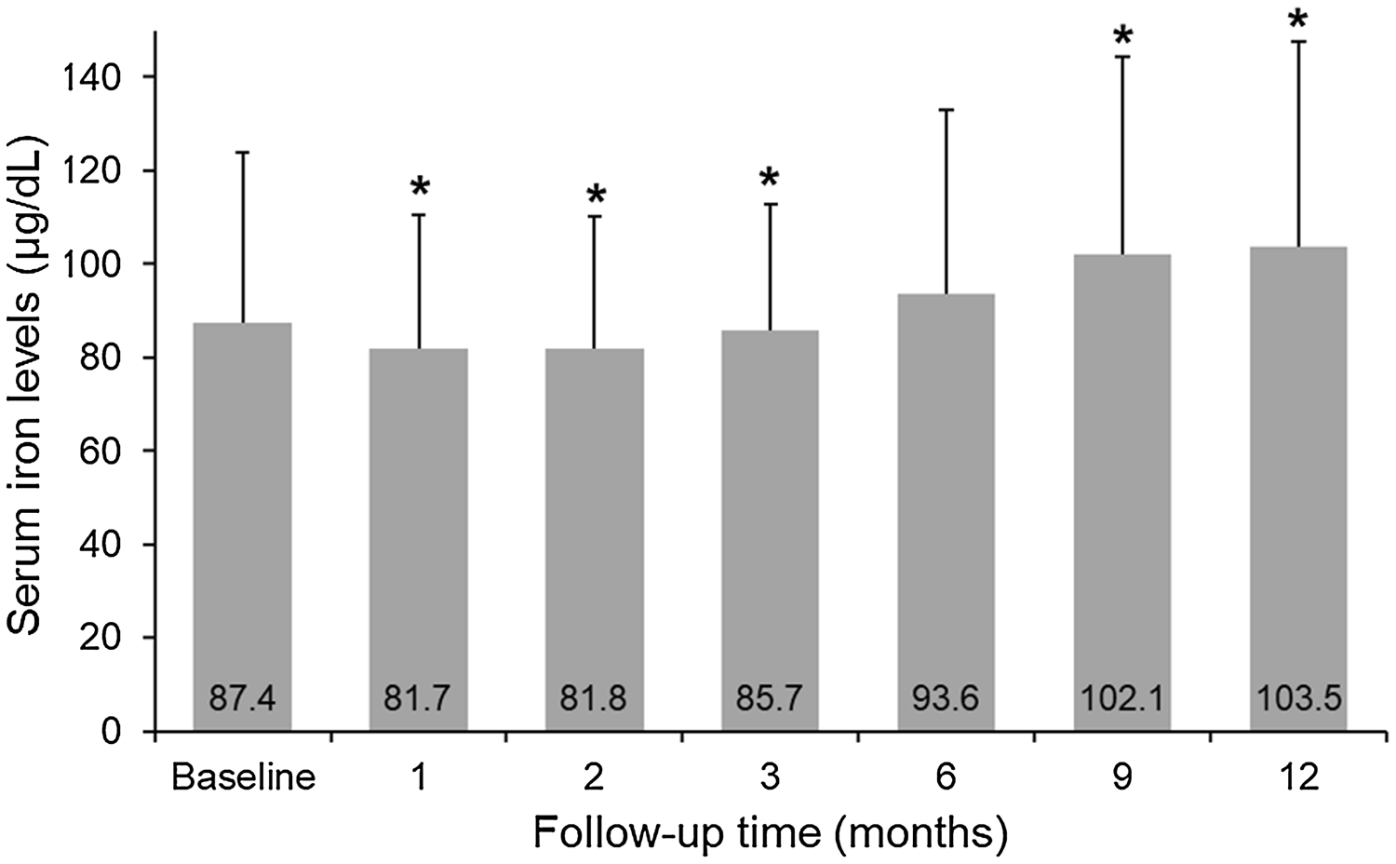
Change in serum iron levels at different timepoints after laparoscopic sleeve gastrectomy.

Table 2 summarizes BMI, serum iron level, kidney function, and metabolic profiles in the recruited patients at postoperative 12 months. BMI significantly decreased from 41.4 ± 5.5 to 28.3 ± 3.6 kg/m^2^ (*p* < 0.001) at postoperative 12 months. After LSG, metabolic profiles and liver function improved (both *p* < 0.05). At postoperative 12 months, serum iron (*p* = 0.004) and folate (*p* = 0.003) levels significantly increased compared with those at baseline. Figure 3 presents the mean serum iron levels at different postoperative timepoints, which shows a J-shaped trend. The percentage of patients with low serum iron levels at postoperative 12 months was 14.7%. No significant difference was noted in eGFR (*p* = 0.864) and serum creatinine level (*p* = 0.942) at postoperative 12 months compared with those at baseline. Figure 1 shows the percentage of patients with various categories of renal function at different postoperative timepoints. The percentage of patients with normal renal function decreased from 73.6% at baseline to 58.1% at postoperative 12 months. Conversely, the percentage of patients with impaired renal function increased from 26.4% at baseline to 32.7% at postoperative 12 months. Glomerular hyperfiltration, which was absent at baseline, was found in 9.2% of the patients at 12 months following surgery.

**Table 2:** Body mass index, kidney function, metabolic profiles and micronutrition at 12-month follow-up.

Variables	Baseline (n = 210)	12-month follow-up (n = 94)	*p* value
Body mass index (kg/m^2^)	41.4 ± 5.5	28.3 ± 3.6	< 0.001
Hemoglobin (mg/dL)	14.2 ± 1.5	13.4 ± 1.8	< 0.001
Serum iron level (µg/dL)	87.4 ± 36.3	103.3 ± 43.7	0.0011
eGFR (mL/min/1.73 m^2^)	98.3 ± 20.3	98.8 ± 22.2	0.864
Creatinine (mg/dL)	0.8 ± 0.30	0.81 ± 0.24	0.942
Glycated hemoglobin, HbA1c (%)	6.6 ± 1.4	5.5 ± 0.5	< 0.001
Fasting glycemia (mg/dL)	105.8 ± 34.0	90.1 ± 12.7	< 0.001
Insulin (IU/mL)	26.1 ± 20.4	7.5 ± 4.5	< 0.001
Total cholesterol (mg/dL)	198.2 ± 34.0	189.0 ± 31.8	0.01
Triglycerides (mg/dL)	190.4 ± 128.8	87.8 ± 39.3	< 0.001
HDL	45.3 ± 9.2	58.8 ± 12.7	< 0.001
LDL	117.8 ± 27.6	105.1 ± 27.3	< 0.001
Vitamin B12 level (pg/mL)	531.7 ± 220.8	496.8 ± 262.8	0.23
Serum Folate level (pg/mL)	6.8 ± 3.6	8.4 ± 4.5	0.003

*eGFR* estimated glomerular filtration rate, *LDL* low-density lipoprotein, *HDL* high density lipoprotein, Data are presented as mean ± standard deviation.

### Associations of serum iron levels with renal function

Figure 2 presents the associations of serum iron levels with renal function (i.e., eGFR) at four timepoints (i.e., baseline and postoperative 1, 6, and 12 months). No correlation was noted between serum iron levels and eGFR at baseline and postoperative 1 and 6 months (Fig. 3A–C), whereas a significant relationship was observed between serum iron levels and postoperative 12-month eGFR (r = 0.224; *p* = 0.03). Additional analysis of the association between serum iron levels and postoperative 9-month eGFR also showed a consistent finding (r = 0.459; *p* < 0.001). No relationship was noted between hemoglobin level and eGFR at the four timepoints (data not shown). Furthermore, no association of eGFR with glucose (r =  − 0.076; *p* = 0.475), HbA1c (r =  − 0.039; *p* = 0.768), and insulin (r =  − 0.127; *p* = 0.449) levels was found at postoperative 12 months. Multiple linear regression analyses using variables including age, sex, postoperative 12-month BMI, %WL, and presence of diabetes revealed that at postoperative 12 months, serum iron levels and presence of diabetes were the independent predictors of eGFR, whereas sex and serum iron levels were found to be the independent predictors for serum creatinine levels (Table 3). No evidence of autocorrelation/collinearity was noted among the six independent variables.

**Table 3:** Multiple regression analyses on associations of independent variables with kidney function (i.e., eGFR and creatinine) at 12-month follow-ups, expressed as standardized effect sizes (β-coefficient).

	eGFR		Creatinine	
	Standardized β-coefficient	*p* value	Standardized β-coefficient	*p* value
Age	− 0.19	0.099	− 0.005	0.965
Gender	0.168	0.138	− 0.688	< 0.001
12-month iron levels	0.242	0.032	− 0.207	0.046
12-month BMI	0.106	0.340	− 0.002	0.981
WL (%)	0.13	0.284	− 0.096	0.351
Diabetes	0.269	0.013	− 0.179	0.067

eGFR, estimated glomerular filtration rate; BMI, body mass index; WL (%), weight loss percentage.

**Figure 4:**
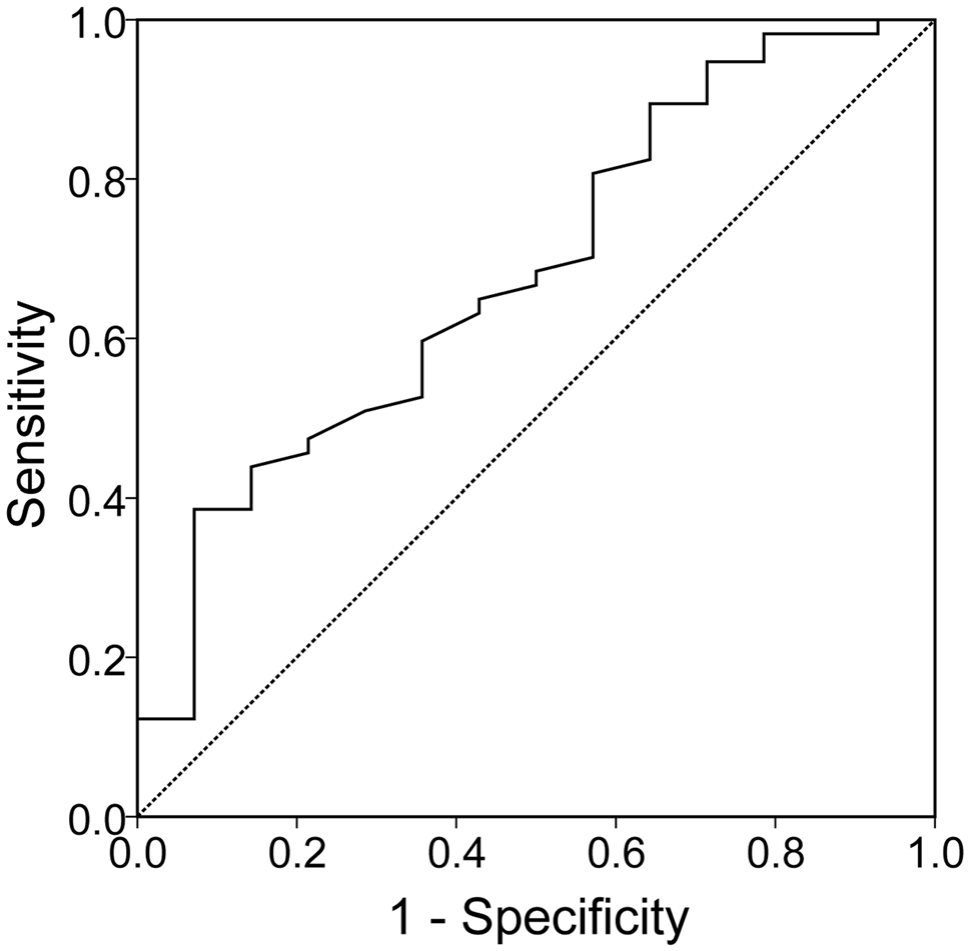
Receiver operating characteristic (ROC) curve showing the ability of serum iron concentrations for predicting normal postoperative kidney function [i.e., estimated glomerular filtration rate (eGFR) ≥ 90 mL/min/1.73 m^2^] in subgroup patients with normal renal function at baseline. The inflection point corresponding to a sensitivity and specificity of 38.6% and 92.9%, respectively. Based on the ROC curve, an ideal cut-off value of 128.5 µg/dL being defined post hoc to identify a target eGFR of ≥ 90 mL/min/1.73 m^2^. The area under curve (AUC) was shown to be 0.682 (95% confidence interval, 0.529–0.836; *p* = 0.035).

Considering that subgroup patients with normal renal function at baseline developed impaired renal function (Fig. 1), an ROC curve was constructed for these patients to determine the point at which the postoperative 12-month serum iron levels could predict a normal postoperative 12-month eGFR (using a target eGFR of ≥ 90 mL/min/1.73 m^2^) with the optimal combined sensitivity/specificity (Fig. 4). Concerning the ROC curve (area under curve, 0.682; 95% confidence interval, 0.529–0.836; *p* = 0.035), an ideal serum iron cutoff of 128.5 µg/dL was defined post hoc to identify the target eGFR. The inflection point corresponded to a sensitivity and specificity of 38.6% and 92.9%, respectively.

## Discussion

Although micronutrient deficiency is common after BS, only few studies have assessed the impact of serum iron levels on renal function after BS. Here, we found that the prevalence of renal impairment was high at postoperative 1 month (i.e., 46.4%), and this prevalence decreased subsequently at postoperative 12 months (i.e., 32.7%) (Fig. 1). Conversely, serum iron levels showed a reverse trend. Serum iron levels increased from 81.7 mg/dL at postoperative 1 month to 103.5 mg/dL at postoperative 12 months. The finding that serum iron levels were positively associated with renal function only at postoperative 9 and 12 months suggested that renal function is influenced by an increase in serum iron levels above a certain threshold. This is the first report to investigate the association between serum iron levels and renal function in patients with MO after BS.

Pooled evidence has demonstrated that BS may enhance kidney function or prevent its further decline in patients with MO^6–8^. Contrariwise, no significant change was noted in postoperative 12-month eGFR compared with that at baseline (98.8 ± 22.2 vs. 98.3 ± 20.3 mL/min/1.73 m^2^; *p* = 0.864) in our study. Furthermore, the percentage of patients with renal impairment increased from 26.4% at baseline to 32.7% at postoperative 12 months, suggesting that some patients postoperatively experience decline in renal function. Our results are consistent with those of two studies that showed that eGFR did not change significantly^9^ or deteriorated at 1 year after BS^10^.

The lack of improvement in eGFR in our patients may be attributed to the surgery type, age, or presence of postoperative acute kidney injury (AKI). First, BS is reported to influence postoperative kidney function; a higher eGFR was noted following gastric bypass than that following sleeve gastrectomy^19^. Because our patients underwent LSG, we cannot exclude the possibility that its effect on postoperative renal function was not obvious. Second, regarding young patients with obesity (mean age of 17 years) receiving BS, eGFR may increase by 3.9 mL/min/1.73 m^2^ for each 10-unit loss of BMI^19^. Because age and obesity are risk factors for CKD, the beneficial effect of BS on improvement in eGFR may not predominate in our relatively aged patients (i.e., mean age, 39.1 ± 10.6 years) whose change in mean BMI was only 13.1 units (from 41.4 to 28.3 kg/m^2^). Third, the prevalence of impaired renal function was high at postoperative 1 month. Previous reports have shown that the risk of AKI in patients with obesity was high after BS^20–22^. Furthermore, it was reported that some patients (about 4.8–10%) with postoperative AKI sustained deterioration in renal function during long-time follow-ups^22,23^. Accordingly, some patients may have experienced AKI and subsequent decline in renal function at postoperative 12 months, leading to no significant change in the overall renal function after BS.

Iron overload may be detrimental to tissues through free radical formation^24,25^. Iron chelation/dietary iron restriction reduced renal damage in animal models of diabetes, renal fibrosis, and CKD^24,25^. However, using experimental rat models, it has been revealed that iron deficiency caused by dietary iron elimination can lead to tissue damage through oxidative stress and mitochondrial damage^26,27^. These results suggest that iron overload and depletion are harmful to tissues. Vaugier et al.^16^ identified serum iron as a significant protective factor for renal allografts. Another study suggests a causal protective effect of circulating and stored iron on renal function in the general population^28^. Although the underlying mechanisms remain unknown, these findings encouraged the investigation of the role of iron in postoperative renal function after BS.

We found that the positive relationship between serum iron concentrations and renal function was obvious at postoperative 9 and 12 months but not at baseline and postoperative 1 and 6 months. Moreover, multiple linear regression demonstrated that serum iron levels and presence of diabetes were predictors of postoperative 12-month eGFR after adjustment for age, sex, postoperative 12-month BMI, %WL, and presence of diabetes. The explanations for the lack of correlation between serum iron levels and renal function at baseline and postoperative 1 and 6 months remain unknown. There are two possible explanations. First, we suggest that the relationship between serum iron level and renal function manifested when serum iron level exceeded a certain threshold. This correlation was obvious only when serum iron levels increased significantly compared with those at baseline. Second, obesity is a state of chronic, low-grade systemic inflammation as evidenced by increased circulating levels of inflammatory proteins including C-reactive protein and proinflammatory cytokines^29^. The pathophysiology of obesity-associated CKD is reportedly associated with renal inflammation^29,30^. Presence of renal inflammation at baseline and early postoperative period (e.g., postoperative 1–6 months) may mask the influence of serum iron level on renal function. When renal inflammation subsided after adequate weight loss, the relationship between iron levels and renal function manifested.

Because of the decreased production of hydrochloric acid in the stomach, reduced gastric capacity, and meat intolerance leading to decreased consumption^31,32^, the postoperative incidence of iron deficiency has been reported to be 30–43% in patients undergoing sleeve gastrectomy^1,15^. In our patients, serum iron level decreased postoperatively, returned to baseline at postoperative 6 months, and then increased at postoperative 9 and 12 months. Moreover, the percentage of patients with low serum iron levels decreased from 21.4% at baseline to 14.7% at postoperative 12 months. The findings of our study were inconsistent with those of some previous studies^1,15^, which may be attributed to several possible explanations. First, during postoperative outpatient appointments, a registered dietitian provided routine standardized nutritional counseling services (e.g., dietetic education and instructions) to all patients. These counseling services may help patients adhere to dietary changes consistent with the surgical procedures that they underwent to reduce the risk of nutritional deficiencies. Moreover, as shown in our results, no other micronutrient deficiencies (e.g., vitamin B12 and serum folate levels) were noted in our patients. Second, obesity-associated chronic inflammation could be a modulator of iron uptake and use. In a previous study involving 178 females with MO scheduled for BS, iron depletion was demonstrated to be significantly associated with elevated levels of inflammatory indices (i.e., C-reactive protein)^33^. Furthermore, BS was associated with a reduction in chronic inflammation and improvement in the serum iron status^33^. Our results regarding the postoperative change pattern of the serum iron status is consistent with the findings of this study^33^.

Anemia is a provoking factor in the progression of end-stage renal failure, and the treatment of anemia by erythropoietin can delay progression of renal failure^24,34^. Accordingly, anemia may be a potential bias in the current study. We further assessed the impact of hemoglobin on renal function at postoperative 12 months, but no association between hemoglobin and eGFR was found.

To correctly interpret our findings, some limitations need to be considered. First, although LSG has obtained popularity as the primary BS at our institute, gastric bypass remains the most prevalent surgery (49.4%) worldwide followed by sleeve gastrectomy (40.7%)^35^. Different technical approaches may lead to various renal outcomes^19^; therefore, our results may not be extrapolated to other surgical populations. Second, the lack of routine blood sampling for the estimation of postoperative serum iron levels and probability of loss to follow-up limited the number of study population. The small number of patients who provided full information for analysis in the current study may limit the reliability of our findings. Other factors including a short follow-up period, substantially different groups of patients at baseline and during postoperative follow-ups, lack of adjustment to potential confounding factors (e.g., iron supplementation) may undermine the conclusions of the current study. Large-scale studies are necessary to support our findings. Third, because of the retrospective nature of the current study, the causal relationship between serum iron levels and renal function cannot be determined. Further, the effects of other potential confounders (e.g., long-term change in blood pressure) on renal function were not assessed because of the lack of relevant data. Fouth, the suggested inflection point, even though very specific, has lower sensitivity, which may pose a limitation in using serum iron as a reliable biomarker in the prognosis of CKD. Finally, the use of eGFR to assess renal function in patients with significant WL has its limitations because subsequent lean body mass changes affect the measurement of serum creatinine level^36^.

## Conclusions

The current report, which is the first for assessing the impact of the serum iron status on kidney function after BS, showed a positive association of serum iron concentrations with renal function at postoperative 12 months but not at baseline or during the early postoperative period. Despite the known relationship between renal function and serum iron levels in the general population, further large-scale studies are necessary to elucidate this causal relationship in patients with obesity and to identify optimal serum iron levels, which can provide the greatest benefit to patients after BS.

## References

[1] Aarts EO, Janssen IM, Berends FJ (2011). The gastric sleeve: losing weight as fast as micronutrients?. Obes. Surg..

[2] Gansevoort RT, Correa-Rotter R, Hemmelgarn BR (2013). Chronic kidney disease and cardiovascular risk: epidemiology, mechanisms, and prevention. The Lancet..

[3] Gansevoort RT, Matsushita K, van der Velde M (2011). Lower estimated GFR and higher albuminuria are associated with adverse kidney outcomes: a collaborative meta-analysis of general and high-risk population cohorts. Kidney Int..

[4] Hall ME, do Carmo JM, da Silva AA, Juncos LA, Wang Z, Hall JE (2014). Obesity, hypertension, and chronic kidney disease. Int. J. Nephrol. Renovasc. Dis..

[5] Garland JS (2014). Elevated body mass index as a risk factor for chronic kidney disease: current perspectives. Diabetes Metab. Syndr. Obes..

[6] Bilha SC, Nistor I, Nedelcu A (2018). The effects of bariatric surgery on renal outcomes: a systematic review and meta-analysis. Obes. Surg..

[7] Zhou X, Li L, Kwong JS, Yu J, Li Y, Sun X (2016). Impact of bariatric surgery on renal functions in patients with type 2 diabetes: systematic review of randomized trials and observational studies. Surg. Obes. Relat. Dis..

[8] Li K, Zou J, Ye Z (2016). Effects of bariatric surgery on renal function in obese patients: a systematic review and meta analysis. PLoS ONE.

[9] Luaces M, Martinez-Martinez E, Medina M (2012). The impact of bariatric surgery on renal and cardiac functions in morbidly obese patients. Nephrol. Dial. Transplant..

[10] Reid TJ, Saeed S, McCoy S, Osewa AA, Persaud A, Ahmed L (2014). The effect of bariatric surgery on renal function. Surg. Obes. Relat. Dis..

[11] Bernert CP, Ciangura C, Coupaye M, Czernichow S, Bouillot J, Basdevant A (2007). Nutritional deficiency after gastric bypass: diagnosis, prevention and treatment. Diabetes Metab..

[12] Kushner RF (2006). Micronutrient deficiencies and bariatric surgery. Curr. Opin. Endocrinol. Diabetes Obes..

[13] Parkes E (2006). Nutritional management of patients after bariatric surgery. Am. J. Med. Sci..

[14] Skroubis G, Sakellaropoulos G, Pouggouras K, Mead N, Nikiforidis G, Kalfarentzos F (2002). Comparison of nutritional deficiencies after Rouxen-Y gastric bypass and after biliopancreatic diversion with Roux-en-Y gastric bypass. Obes. Surg..

[15] Alexandrou A, Armeni E, Kouskouni E, Tsoka E, Diamantis T, Lambrinoudaki I (2014). Cross-sectional long-term micronutrient deficiencies after sleeve gastrectomy versus Roux-en-Y gastric bypass: a pilot study. Surg. Obes. Rel. Dis..

[16] Vaugier C, Amano MT, Chemouny JM (2017). Serum iron protects from renal postischemic injury. J. Am. Soc. Nephrol..

[17] Bohne F, Martinez-Llordella M, Lozano JJ (2012). Intra-graft expression of genes involved in iron homeostasis predicts the development of operational tolerance in human liver transplantation. J. Clin. Invest..

[18] Levey AS, Stevens LA, Schmid CH (2009). A new equation to estimate glomerular filtration rate. Ann. Intern. Med..

[19] Nehus EJ, Khoury JC, Inge TH (2017). Kidney outcomes three years after bariatric surgery in severely obese adolescents. Kidney Int..

[20] Ahluwalia JS, Chang PC, Tai CM, Tsai CC, Sun PL, Huang CK (2016). Comparative study between laparoscopic adjustable gastric banded plication and sleeve gastrectomy in moderate obesity–2 year results. Obes. Surg..

[21] Thakar CV, Kharat V, Blanck S, Leonard AC (2007). Acute kidney injury after gastric bypass surgery. Clin. J. Am. Soc. Nephrol..

[22] Sharma SK, McCauley J, Cottam D (2006). Acute changes in renal function after laparoscopic gastric surgery for morbid obesity. Surg. Obes. Relat. Dis..

[23] Nor Hanipah Z, Punchai S, Augustin T, Brethauer SA, Schauer PR, Aminian A (2018). Impact of early postbariatric surgery acute kidney injury on long-term renal function. Obes. Surg..

[24] Łukaszyk E, Łukaszyk M, Koc-Żórawska E, Tobolczyk J, Bodzenta-Łukaszyk A, Małyszko J (2015). Iron status and inflammation in early stages of chronic kidney disease. Kidney Blood Press. Res..

[25] Ikeda Y, Ozono I, Tajima S (2014). Iron chelation by deferoxamine prevents renal interstitial fibrosis in mice with unilateral ureteral obstruction. PLoS ONE.

[26] Walter PB, Knutson MD, Paler-Martinez A (2002). Iron deficiency and iron excess damage mitochondria and mitochondrial DNA in rats. Proc. Natl. Acad. Sci. USA..

[27] Knutson MD, Walter PB, Ames BN, Viteri FE (2000). Both iron deficiency and daily iron supplements increase lipid peroxidation in rats. J. Nutr..

[28] Del Greco MF, Foco L, Pichler I (2016). Serum iron level and kidney function: a Mendelian randomization study. Nephrol. Dial. Transplant..

[29] Fenske WK, Dubb S, Bueter M (2013). Effect of bariatric surgery-induced weight loss on renal and systemic inflammation and blood pressure: a 12-month prospective study. Surg. Obes. Relat. Dis..

[30] Bonnet F, Deprele C, Sassolas A (2001). Excessive body weight as a new independent risk factor for clinical and pathological progression in primary IgA nephritis. Am. J. Kidney Dis..

[31] von Drygalski A, Andris DA (2009). Anemia after bariatric surgery: more than just iron deficiency. Nutr. Clin. Pract..

[32] Shankar P, Boylan M, Sriram K (2010). Micronutrient deficiencies after bariatric surgery. Nutrition.

[33] Anty R, Dahman M, Iannelli A (2008). Bariatric surgery can correct iron depletion in morbidly obese women: a link with chronic inflammation. Obes. Surg..

[34] Gouva C, Nikolopoulos P, Ioannidis JP, Siamopoulos KC (2004). Treating anemia early in renal failure patients slows the decline of renal function: a randomized controlled trial. Kidney Int..

[35] Welbourn R, Pournaras DJ, Dixon J (2018). Bariatric surgery worldwide: baseline demographic description and one-year outcomes from the second IFSO Global Registry Report 2013–2015. Obes Surg..

[36] Chagnac A, Weinstein T, Herman M, Hirsh J, Gafter U, Ori Y (2003). The effects of weight loss on renal function in patients with severe obesity. J. Am. Soc. Nephrol..

